# Analysis of APC mutation in human ameloblastoma and clinical significance

**DOI:** 10.1186/s40064-016-1904-3

**Published:** 2016-03-10

**Authors:** Ning Li, Bing Liu, Chengguang Sui, Youhong Jiang

**Affiliations:** Department of Tumor Biotherapy and Cancer Research, The First Affiliated Hospital of China Medical University, Shenyang, 110001 Liaoning Province China; The 202nd Hospital of PLA, Shenyang, 110003 Liaoning Province China

**Keywords:** Wnt/β-catenin, Ameloblastoma, APC, Mutation

## Abstract

As a highly conserved signaling pathway, Wnt/β-catenin signal transduction pathway plays an important role in many processes. Either in the occurrence or development of tumor, activation of this pathway takes an important place. APC inhibits Wnt/β-catenin pathway to regulate cell proliferation and differentiation. This study aimed to investigate the function of cancer suppressor gene. PCR amplification and sequencing method was used to analyze APC mutations of human clinical specimens. The pathological specimens were collected for PCR and clear electrophoretic bands were obtained after electrophoresis. The gene sequence obtained after purification and sequencing analysis was compared with the known APC gene sequence (NM_000038.5). Base mutations at APC 1543 (T → C), APC-4564 (G → A), APC-5353 (T → G), APC-5550 (T → A) and APC-5969 (G → A) locus existed in 22 (27.5 %), 12 (15 %), 5 (6.25 %), 13 (16.25 %) and 12 patients (15 %), respectively. Gene mutations existed in ameloblastoma, and the mutation loci were 1543 locus (T → C), 4564 locus (G → A), 5353 locus (T → G), 5550 locus (T → A) and 5969 locus (G → A) 15 %, respectively. APC mutation plays a certain role in monitoring the tumor malignant degree as it may indicate the transition process of ameloblastoma malignant phenotype.

## Background

Ameloblastoma (AB) is the most common benign odontogenic and epithelium originated tumor in jaw. In China, AB takes up to 36 % of odontogenic tumors. The tumor mainly consists of ameloblastic structures without enamel and other solid tissues. Most of the tumors occur in jaws, leading to expansion of jaws and facial deformations. Although AB is a benign tumor, it grows invasively with a high recurrent rate after surgery. Besides, there are a few reports of malignant transformation or even distant metastasis. In 2005, WHO has classified ABs into four categories, solid/multicystic type, outside the bone/peripheral type, desmoplastic type and unicystic type. All these types showed differences in aspects of age, location, imaging and clinical prognosis. Excochleation was used in treatments of ABs with a tendency to recurrence, thus extended resection is mainly adopted. In consideration of lacking effective therapeutic methods, this study aimed to investigate the mechanism of ABs to look for new targets of treatments, having significance in prevention and treatments of ABs.

Recnetly, there are many studies about ABs, especially on its molecular biological mechanism. AB is a tumor related to tooth development, while Wnt signaling pathway is inevitable during tooth development, thus it is speculated that Wnt signaling pathway might be associated with genesis of AB. Expression of downstream factors in Wnt signaling pathway in ABs was detected in a series of studies previously. Results of in situ hybridization showed that mRNA of c-myc and cyclin D1 was strongly expressed, and their positive expression increased with recurrences and malignant transformation. Results of immunohistochemistry showed that VEGF and mmp7 had high positive expression in ABs. Wnt signaling pathway has been confirmed with a significant role in genesis of ABs.

In recent years, the study of cancer suppressor gene APC is paid more and more attention to in-depth study of tumor. APC, a multifunctional cancer suppressor gene, is involved in not only Wnt signal pathway to regulate β-catenin degradation (Patel et al. 2015), but also regulating cytoskeleton movement, controlling cell cycle and influencing cell migration and division (Eom et al. 2014). APC gene mutations would cause regulatory function disorder, which is closely associated with occurrence and development of tumor.

As a highly conserved signaling pathway, Wnt/β-catenin (Logan and Nusse 2004; Sparks et al. 1998; Miyoshi et al. 1998) signal transduction pathway plays important roles in many processes. Either in the occurrence or development of tumor, activation of this pathway takes an important place. APC is able to regulate cell proliferation and differentiation by inhibiting Wnt signal pathway. APC deficiency can induce β-catenin accumulation in the nucleus to lead to the activation of the transcription factors TCF and LEF, thus activating the classical Wnt/β-catenin/Tcf signal pathway. In the gastrointestinal endocrine tumor, activated Wnt signal pathway would result in β-catenin aggregation in the nucleus. Meanwhile, the invasiveness of the tumor is relevant to the expression quantity of β-catenin in the nucleus. Galiatsatos and Foulkes (2006) discovered that in mice with mutative truncated APC protein, intestinal tumors could develop spontaneously. Therefore, studying the function of the suppressor gene APC will have an important significance in learning the molecular mechanism of the occurrence and development of ameloblastoma.

Tumorogenesis is caused by the genetic changes. Specifically, due to some causes, the proto-oncogene is activated and cancer suppressor gene is inactivated so as to induce abnormal genetic expression, finally leading to tumorogenesis (Rubinfeld et al. 1997; Sanders et al. 1998; Hashizume et al. 1996). The proto-oncogene is activated through genetic point mutation, gene amplification and promoter chromatid recombination, while the expression of the cancer suppressor gene is silenced due to gene mutation, gene deficiency and promoter methylation, thereby, inhibiting its activation. Therefore, the influence of APC gene changes on the occurrence of ameloblastoma is considered from genetics mechanism and epigenetic mechanism. Namely, the former thinks that the point mutations are formed by the changing DNA nucleotide sequence, and the latter means that gene expression is influenced through DNA chemical modification from the transcriptional level, including changes in the levels of gene methylation and acetylation, rather than changes in DNA coding sequence. According to reports of Sekine, Kawabata, Miyake, Siriwardena and Tanahashi (Tanahashi et al. 2008; Kumamoto and Ooya 2005; Siriwardena et al. 2009; Sekine et al. 2003; Kumamoto et al. 2001), 64 cases of Abs were analyzed. Among them, only 3 cases (4.7 %) having CTNNB1 mutations, however cases with malignant did not have CTNNB1 mutations. Thus, there are few β-catenin mutations among ABs, which indicated that accumulation of β-catenin in cytoplasm had no relation to its mutations. Some researchers speculated that accumulation of β-catenin in cytoplasm might be caused by abnormalities of other molecules, and believed that abnormalities of GSK-3β, APC and Axin in degradation compounds might be a factor of an abnormal accumulation of β-catenin. At present, there are still few researches about degradation compounds. Among them, GSK-3β in Abs has not been reported, while research results of expression and mutation of APC were not consistent with each other. Therefore, this study aimed to investigate the function of cancer suppressor gene APC is of significance in learning the molecular mechanism of occurrence and development of ameloblastoma.

## Results

### Expression of β-catenin

**Fig. 1:**
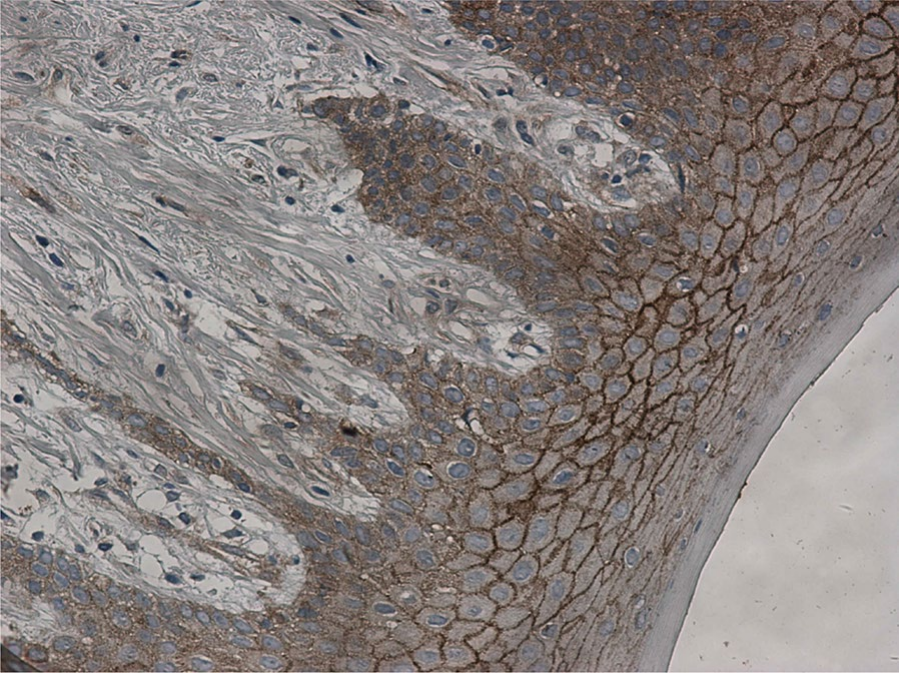
β-catenin is expressed in the normal oral mucous membrane, with no aberrant expression in cytoplasm and nucleus, by SP ×20

**Fig. 2:**
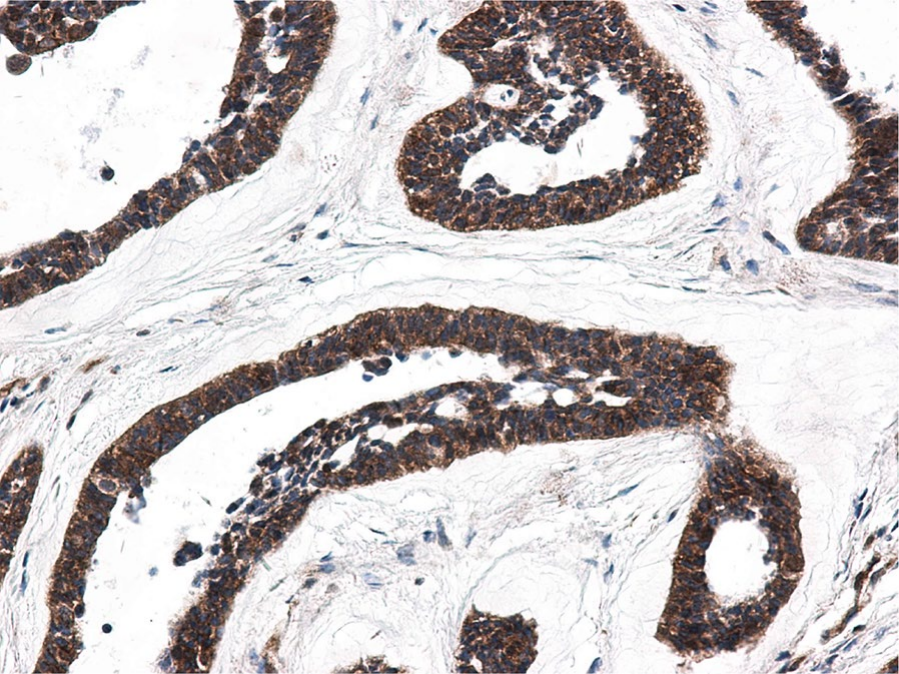
β-catenin is aberrant expressed in cytoplasm of ABs, by SP ×20

**Fig. 3:**
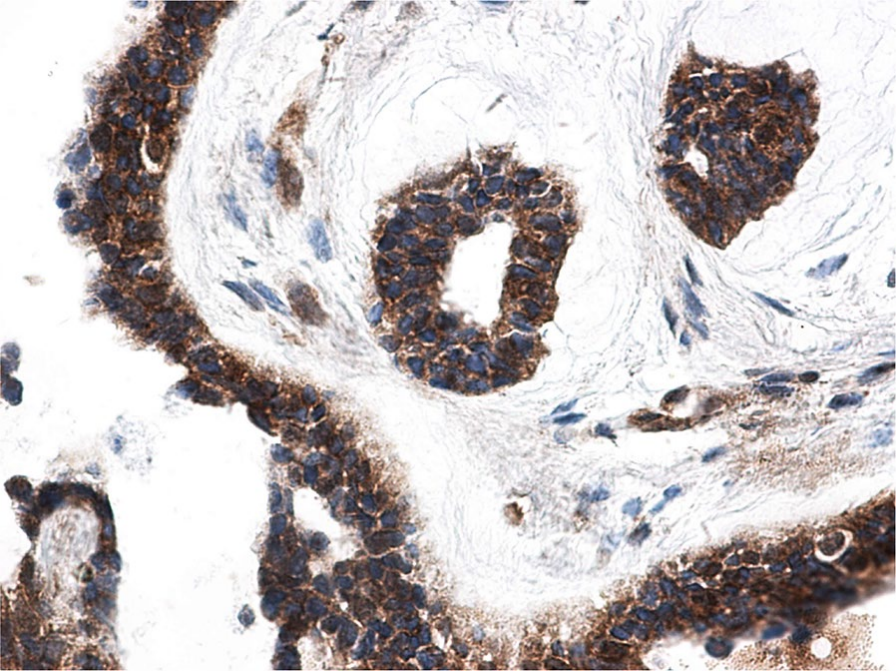
β-catenin is aberrant expressed in nucleus of ABs (*arrow*), by SP ×40

Expression of β-catenin was claybank in normal oral mucosa, with positive expression locating on cell membrane. There was no abnormal expression in cytoplasm (Fig. 1). While in ABs, there was abnormal expression in cytoplasm (77 cases, 96.3 %) and nucleus (24 cases, 30 %), and the expression on cell membrane decreased. All cases with abnormal expression in nucleus showed abnormal expression in cytoplasm (Figs. 2, 3).

### Detection results of APC mutation

**Fig. 4:**
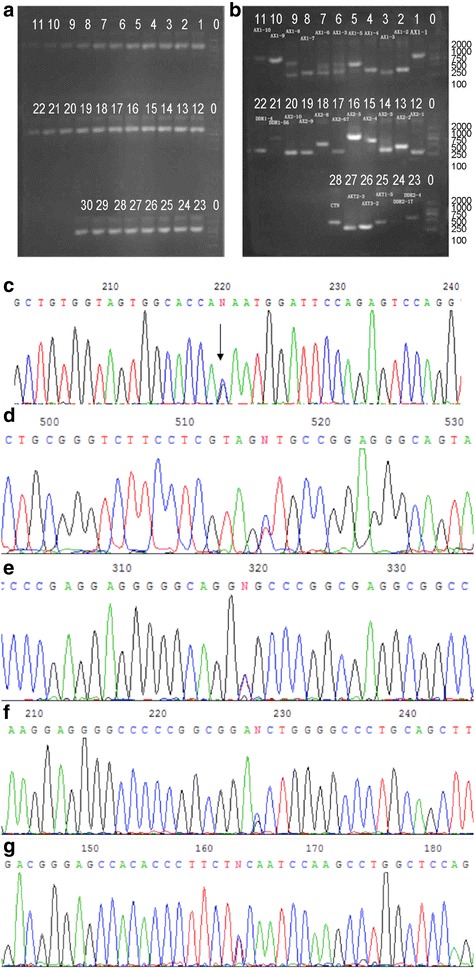
Agarose electrophoresis and sequencing results for specimen mutation. **a** 0: DL2000; 1–30: sample 1–30, **b** 0: DL2000; 1–5: AX1-1–5; 6: AX1-3; 7–11: AX1-6–10; 12–16: AX2-1–5; 17: AX2-67; 18–20: AX2-8–10; 21: DDR1-56; 22: DDR1-4; 23: DDR2-4; 23: DDR2-17; 25: AXT1-3; 26: AXT3-2; 27: AXT2-3; 28: CTN, **c** base mutation at 37 codon: serine → cysteine, **d** serine → asparagine; base mutation at 496 codon: alanine → threonine in sample 13, **e** base mutation at 603 codon: alanine → proline, **f** base mutation at 738 codon: serine → phenylalanine, **g** base mutation at 50 codon: proline → serine

The pathological specimens were collected for PCR and clear electrophoretic bands were obtained after electrophoresis (Fig. 4). The gene sequence obtained after purification and sequencing analysis was compared with the known APC gene sequence (NM_000038.5), and the results showed that base mutation at APC 1543 (T → C), APC-4564 (G → A), APC-5353 (T → G), APC-5550 (T → A) and APC-5969 (G → A) locus existed in 22 (27.5 %), 12 (15 %), 5 (6.25 %), 13 (16.25 %) and 12 patients (15 %), respectively (Tables 1, 2).

**Table 1:** Detection and analyses results of APC locus mutation

Mutation locus	Number of cases	Mutation	Mutation rate (%)
1543	22	T → C	27.5
4564	12	G → A	15
5353	5	T → G	6.25
5550	13	T → A	16.25
5969	12	G → A	15

**Table 2:** Base changes with tumor subtypes

Gene	Sample (n)	Age		Gender		Location			Type			Pathological type						
		<50	>50	M	F	Upper	Lower	Other	Primary	Recurrent	Malignant	Follicular	Acanthomatous	Plexiform	Desmoplastic	Peripheral	Mixed	Single cystic
1543	22	10	12	14	8	2	20		16	6		12	3	4	1	3		1
4564	12	6	6	7	5	1	10	1	11	1		8	1	1		1		1
5353	5	3	2	3	2	1	4		4	1		4	1					
5550	13	7	6	8	5		12	1	11	2		9		3		2		
5969	12	5	7	5	7	1	11		10	2		9	1	1	1			

Notes: M: male; F: female

### Analysis results of methylation

After DNA denaturation, with the treatment of sodium hydrogen sulfite, unmethylated C could be efficiently transformed into U, while the methylated C remained unchanged. After the PCR amplification, the unmethylated C would be finally transformed into T, while the methylated C was still C. PCR amplification was conducted, and the amplification products were sequenced after detected with agarose gel electrophoresis. Afterwards, the sequencing results were analyzed. Through comparison, a good correspondence to the original sequence before transformed with sodium hydrogen sulfite was shown (Table 3).

**Table 3:** Detection results of APC methylation

APC	2	3	4	5	8	9	10	15	17	21
CPG1	U	U	U	U	U	U	U	U	U	U
CPG2	U	U	U	U	U	U	U	U	U	U
CPG3	U	U	U	U	U	U	U	U	U	U
CPG4	U	U	U	U	U	U	U	U	U	U
CPG5	U	U	U	U	U	U	U	U	U	U
CPG6	U	U	U	U	U	U	U	U	U	U
CPG7	U	U	U	U	U	U	U	U	U	U
CPG8	U	U	U	U	U	U	U	U	U	U
CPG9	U	U	U	U	U	U	U	U	U	U
CPG10	U	U	U	U	U	U	U	U	U	U
CPG11	U	U	U	U	U	U	U	U	U	U
CPG12	U	U	U	U	U	U	U	U	U	U
CPG13	U	U	U	U	U	U	U	U	U	U
CPG14	U	U	U	U	U	U	U	U	U	U
CPG15	U	U	U	U	U	U	U	U	U	U
CPG16	U	U	U	U	U	U	U	U	U	U
CPG17	U	U	U	U	U	U	U	U	U	U
CPG18	U	U	U	U	U	U	U	U	U	U
CPG19	U	U	U	U	U	U	U	U	U	U
CPG20	U	U	U	U	U	U	U	U	U	U
CPG21	U	U	U	U	U	U	U	U	U	U
CPG22	U	U	U	U	U	U	U	U	U	U

As to the original sequence (380 bp and 22 CpG locus) in APC promoter region, the results showed that in the 30 ABs cases, no methylation occurred. This indicated that the changes of methylation level in APC promoter region have not been found among the collected specimens of clinical ameloblastoma tissues.

## Discussion

APC mutation mainly takes place in the high incidence area of mutation in the fifteenth exon of APC gene, thus forming the truncated APC protein at C terminal domain. The change of APC gene can not only induce familial adenomatous polyposis, but also is related to the early occurrence of colorectal cancer. It exists in the over 85 % of sporadic colorectal cancer (Nathke 2004).

APC gene family includes APC, APC-L/2, H-APC, D-APC, E-APC and other congeners, and APC is first discovered and more common. APC gene locates in chromosome 5q21 (Behrens 2005), and a series of analyses of its cDNA clone show an open reading frame of 8535 bp. There are 21 exons, and the fifteenth one is 6571 bp, also the largest one, accounting for more than 75 % in this coding region. APC gene can code a large protein molecule with the molecular weight of 300 kD consisting of 2843 amino acids, which mainly plays a supporting role. It is also known as APC protein. N terminal domain of wild-type APC includes several functional structural domains, and there a very high homology among different species. N-terminal domain contains multiple repeating regions with hydrophobic residues which regulate the formation of APC homodimer, followed by 7 conserved Armadillo repeating regions which play a very important role on the interaction among proteins and cell adhesion. The proteins combined with this region are mainly PP2A, Asef, Kap3 and Axin1 (Peifer and Polakis 2000). The intermediate region of APC molecule consists of 3–4 repeating regions made of 15 amino acid residues and 7 repeating regions made of 20 amino acid residues. These regions play an important role in the Wnt signal pathway through interaction with other proteins. 3 amino acid sequences called “SAMP” exist among the 20 amino acid repeating regions and involve the binding site between Axin and APC. APC proteins discovered currently are able to participate in Wnt pathway and interact with β-catenin, Axin protein and Gsk 3β. Therefore, APC gene mutation can directly influence the function of Wnt signal pathway and the interaction between Gsk 3β and β-catine to take part in the tumor occurrence (Jones and Baylin 2007; Goel et al. 2007; Kim et al. 2003; Garcia-Manero et al. 2002).

Chromosome instability (CIN) is considered as an important factor promoting tumorogenesis (He et al. 1998; Shen et al. 2002; Wajed et al. 2001). In colon cancer and invasive endocrine tumor cells induced by APC mutation, CIN is very distinct (Rubinfeld et al. 1996). In the mitosis, a vital function of microtubule is to participate in the formation of spindle and divide equally the chromosome between the two daughter cells. APC protein, positioning in centromere, spindle and centrosome directly, takes part in the normal spindle arrangement in equatorial plate, centromere stability and regulation of the connection between spindle microtubule and chromosome. A study by Aoki et al. (2007) showed that APC mutation would induce error separation of chromosome. In the embryonic stem cell and intestinal polyp cells of mice, the truncated APC mutation may cause chromosomal aberration and non-integer value, thus leading to the chromosome instability. Fodde (2003) found that in the HCT116 cell strain of human colon cancer cells expressing the overall length APC, although the C terminal domain of APC (including microtubule binding domain) could be expressed, the none-integer chromosome was also developed. Moreover, in HCT116 cell, the expression of APC N terminal domain may affect the interaction between centromere and microtubule to induce CIN. Therefore, APC was thought to affect the centromere function and chromosomes separation through its interaction with microtubule. The results of this study indicated that there were APC gene mutations in the occurrence process of ameloblastoma, which mainly manifested as point mutation. Thereby, the amino acid at individual site changed in APC. If this change affected the structure and functions of APC protein, the stability of APC protein complex would be affected to change the degradation of complex to β-catenin and promote occurrence of certain malignant phenotype (invasiveness and metastasis) in ameloblastoma.

Change of abnormal DNA methylation is the third mechanism leading to expression inactivation of cancer suppressor gene (Wajed et al. 2001). When cells are in embryonic and multipotent stem cells periods, the whole genome showed hypomethylation. Namely, cells are undifferentiated and exuberantly proliferated. Moreover, with age (except of germ cells), DNA methylation level of cells is gradually increasing, and a large number of gene transcriptions are suppressed so that the activation of cell proliferation is gradually decreased and cells are getting into the differentiation–maturity-senility process. In mammalian cells, extensive hypomethylation and hypermethylation of gene promoter specific region CpG island often occur (Das and Singal 2004). Recent studies suggested that extensive hypomethylation is able to activate proto-oncogene and lead to CIN (Gaudet et al. 2003). Besides, increased expression of oncogene because of their hypomethylation status is also a mechanism promoting tumorogenesis. For example, among adult male mice exposed to inorganic arsenic in embryonic period, estrogen receptor (ER2α) gene and Cyclin D1 gene in the livers exhibited hypomethylation, resulting in an overexpression of ER2α. This indicated a possible correlation with hepatocellular carcinoma (Waalkes et al. 2004). A study by Gaudet et al. (2003) showed that there may be three mechanisms of hypomethylation inducing tumors: (a) hypomethylation induced generation of endogenous retrovirus substances that could activate the proto-oncogene. (b) hypomethylation could activate the proto-oncogene through epigenetic effects; (c) hypomethylation can bring about genomic instability. The specific region with hypermethylation, generally, is the CpG island crossing housekeeping gene and cancer suppressor gene promoter. Furthermore, hypomethylation helps the tumor development due to the ability of inducing mutation of coding region or gene inactivation. At present, the genes which had been identified to have a high risk of hypermethylation in various tumors include those involved in DNA repair, cell cycle, cell differentiation, cell apoptosis, formation of drug resistance, tumor metastasis, tumor infiltration and angiogenesis (Wang et al. 2009). In human genes, about half of gene promoter regions are rich in CpG island, which are prone to methylation and gene silencing. Moreover, some genes are methylated more easily than other genes. Apart from the genes positioning in the inactivated X chromosome and imprinted genes, the vast majority of CpG islands are non-methylated under the normal physiological conditions, while they are over methylated in tumor tissues. This methylation often induces gene silencing, which is continuous with age. Different from gene mutation, the methylated DNA nucleotide sequence is not changed and gene transcription is affected merely through modification of individual bases. So, methylation is a reversible process. However, our experiments results suggested that no gene methylation occurred in APC gene promoter region of ameloblastoma tissues. Therefore, it was considered that, in the occurrence process of ameloblastoma, changes of APC gene took place in DNA level (i.e., changes in genetics). Meanwhile, the point mutations were formed by changes of DNA nucleotide sequence, rather than epigenetic mechanism which means that changes after gene modification would lead to mRNA changes in gene transcription level. APC gene locus mutations may be related to appearance of some malignant phenotypes in ameloblastoma, which may provide the basis for assessing grade malignancy of ameloblastoma and judging the prognosis in clinic.

## Conclusions

APC gene mutation exist in ABs, and the mutation loci are 1543 locus (T → C), 4564 locus (G → A), 5353 locus (T → G), 5550 locus (T → A) and 5969 locus (G → A), respectively, with the mutation rates of 27.5, 15, 6.25, 16.25 and 15 %, respectively.

No gene methylation changes exist in APC gene promoter region of ameloblastoma tissues. Therefore, it is considered that, in the occurrence process of ameloblastoma, changes of APC gene occur in DNA level (i.e., changes in genetics). Meanwhile, the point mutations are formed by changes of DNA nucleotide sequence, rather than epigenetic mechanism which means that changes after gene modification would lead to mRNA changes in gene transcription level.

## Methods

### Clinical pathologic specimens

This study has been approved by the Ethics Committee of The First Affiliated Hospital of China Medical University. Thirty cases of fresh ameloblastoma (Abs) tissues were obtained from 2004 to 2009 in the surgical department in Hospital of Stomatology of China Medical University. In control group, 5 cases of NOM were obtained through wisdom teeth removal in the stomatology clinic of this hospital, and 3 cases of dental germ tissues were from abortus in obstetrical department of People’s Liberation Army No. 202 Hospital. Informed consent was obtained from the patients for all specimens. Besides, the excised specimens were collected immediately during the surgery, and the specimens were stored at −86 °C for inspection after quick freezing in liquid nitrogen. All specimens were classified referring to WHO classification criteria in 2005. Among these 30 cases [16 males and 14 females; mean age: 44 (16–72 years)] included 16 cases of primary ABs and 12 cases of recurrent ABs (relapse after scaling in 9 cases and relapse after removal in 4 cases, with the relapse interval ranging from 4 months to 14 years, and malignant Abs in 2 cases). The peak ages of this disease were 32–49 years old, accounting for 36.7 % (11/30). 24 cases occurred in the lower jawbone, accounting for 80.0 % (24/30), including 9 cases in the left lower jawbone, 9 cases in the right lower jawbone and 6 cases in mandibular symphysis. 3 cases occurred in the upper jawbone, accounting for 10.0 % (3/30), including 2 cases involving the left upper jawbone and 1 case involving the right jawbone. Moreover, 1 case was found in gingiva, 1 case in the parapharyngeal space of the left palate, and 1 case in the left cheek. Abs were classified according to pathology, and solid/polycystic Abs accounted for 83.3 % (25/30), single cystic ABs 3.3 % (1/30), desmoplastic ABs 3.3 % (1/30), and peripheral ABs 10 % (3/30), respectively. Furthermore, 25 solid Abs included 14 cases of follicular ABs, 5 cases of plexiform ABs, 3 cases of acanthomatous ABs, 2 cases of basal cell ABs and 1 case of follicular mixed with plexiform Abs (Table 4).

**Table 4:** Patient demographics

Sample	Age	Gender	Location	Primary/recurrent/malignant	Pathological type
1	26	Male	Lower	Primary	Single cystic
2	81	Male	Lower	Recurrent	Follicular
3	54	Female	Parapharyngeal space	Primary	Peripheral
4	34	Male	Lower	Primary	Follicular
5	53	Female	Lower	Recurrent	Follicular
6	46	Male	Lower	Recurrent	Acanthsomatous
7	40	Female	Lower	Primary	Follicular
8	69	Female	Cheek	Recurrent	Peripheral
9	21	Male	Lower	Primary	Plexiform
10	59	Male	Lower	Primary	Follicular
11	45	Male	Lower	Primary	Follicular
12	43	Female	Upper	Primary	Follicular
13	59	Female	Gingiva	Recurrent	Peripheral
14	46	Male	Lower	Primary	Plexiform + Follicular
15	40	Female	Lower	Recurrent	Follicular
16	72	Male	Lower	Primary	Desmoplastic
17	31	Male	Lower	Recurrent	Follicular
18	16	Female	Lower	Recurrent	Plexiform
19	45	Female	Lower	Primary	Follicular
20	40	Female	Upper	Recurrent	Follicular
21	56	Male	Lower	Recurrent	Follicular
22	18	Male	Lower	Primary	Follicular
23	21	Female	Lower	Primary	Follicular
24	22	Female	Lower	Primary	Follicular
25	44	Male	Lower	Recurrent	Basal
26	59	Female	Lower	Primary	Acanthomatous
27	68	Male	Lower	Malignant	Basal
28	26	Male	Lower	Primary	Basal
29	58	Male	Lower	Primary	Plexiform
30	40	Female	Upper	Malignant	Plexiform

### Genomic extraction reagent

Phenol, Chloroform and Isoamyl alcohol (25:24:1 V/V). SNET: 20 mM Tris–Cl (pH8.0); 5 mM EDTA (pH8.0); 400 mM NaCl; 1 % (m/V) SDS; TE: 10 mM Tris–Cl (pH8.0); 1 mM EDTA (pH8.0); Protease K (20 mg/ml); Lysis buffer; adding protease K into SNET until the final concentration was 400 μg/ml.

### PCR reagent

Taq enzyme was made from Beijing Sunbiotech co., Ltd; dNTP was made from Beijing Sunbiotech co., Ltd; All primers were synthesized by Beijing Sunbiotech co., Ltd; Betaine was purchased from Sigma (Table 5).

**Table 5:** Primers of APC exon

APC exon	Primer	Sequence	Gragment (bp)	Temperature (°C)	Time (sec)
1	APC-1F	TTTTGTTTCCTTTACCCCTTTC	450	55	30
	APC-1R	CCATTGGAGTTTTACACTTATTTTC			
2	APC-2F	AATCACCATTATCTCAAAATATCAC	411	55	30
	APC-2R	GCTAACATACCAAGAAAACTGAT			
3	APC-3F	AACACACTCCTTATTTTTACCCT	516	55	30
	APC-3R	ATGCAAAATGCTCTAAGTGTTAG			
4	APC-4F	TAACAACTGATGTAAGTATTGCTCT	470	57	30
	APC-4R	TAGTTGCCTAGTTGAACCCTG			
5	APC-5F	ATTGATACTTTTTTATTATTTGTGG	400	57	30
	APC-5R	CATCTAACTCTGTCTCTCCCTTA			
6	APC-6F	TGAGAATGATTTGACATAACCCTGA	227	57	30
	APC-6R	ATACCCACAAACAAGAAAGGCA			
7	APC-7F	ACTTCACTTTCCCCTTACCGAG	400	57	30
	APC-7R	TCTTAGAACCATCTTGCTTCATACT			
8	APC-8F	ATTCCTTCAATGCTTTTCATCA	400	57	30
	APC-8R	CCAGGGTTTGTAATCTTCTGTTA			
9	APC-9F	TTGTTTCTAAACTCATTTGGCC	525	55	30
	APC-9R	TGATATGAATTTTCTCCTCTTAGTC			
10	APC-10F	TTGTAGTATTTATTCATCCTTTCAG	471	55	30
	APC-10R	AGGGTAGCAGTTTCTGTGATTC			
11	APC-11F	TGGCATAAAATGGAATAATTGTCAG	500	55	30
	APC-11R	AGTAAAGATAAGCGAATGTGAAGCA			
12	APC-12F	ATTGATTCCATCCAAATAAGAGG	470	55	30
	APC-12R	TGAGCTGAGATTGCACAACTG			
13	APC-13F	AGAAGGTCTTGAACTCCTGGT	400	55	30
	APC-13R	AGAAATTAGGAAATCTCATGGC			
14	APC-14F	AGTGAGGGACGGGCAATAGGATA	576	55	30
	APC-14R	ACCTATGGGCTACACCTCTCAACTA			
15	APC15-1F	TATGCCTTTTGTCTTCTATCCTTTT	1282	57	45
16	APC15-1R	GTCTTGCCCATCTTTCATTCTGT			
	APC15-11R	AACCATCAAGAGTGCCTCCCAAAA			

### Detection of APC gene mutation

PCR amplification was used for direct sequencing, and phenol–chloroform extraction was adopted. According to the instructions of operating manual, genome DNA was extracted. Primer 3 software was adopted to design primer sequence for APC by Beijing Sunbiotech co., Ltd. PCR amplification and agarose gel electrophoresis were conducted. Detection and data collection were finished by ABI 3730. Gene was compared based on http://blast.ncbi.nlm.nih.gov/Blast.cgi. Amplified PCR products were sequenced using PCR primers with the assistance of GeneMapper4.0 software.

### Methylation detection of APC gene

Sequencing after treated with NaHSO_3_ adopted the principle that the unmethylated cytosine could be deaminized to form uracil by sodium hydrogen sulfite, thus achieving sequencing after amplified with specific primers. The original sequences (380 bp and 22 CpG loci) in the APC promoter region were predicted by bioinformatics. According to kit instructions, genome DNA of diaphragm tissues was adopted and was purified by Wizard Clean-up system of America Promega Corporation after incubated for 16 h in sodium hydrogen sulfite at 55 °C. After ending modification with NaOH, these genome DNAs were suspended in the double distilled water. With the assistance of primer 5 software, the primer of the modified DNA specimens was designed for primer amplification and primer sequencing (Table 6). The promoter region of APC gene to be detected, PCR reaction, purification of Agarose gel recovered fragments, ligation reaction, transformation, positive cloning identification, upstream primer and downstream primer were sequenced and verified by Invitrogen Corporation.

**Table 6:** Primer sequence detection of promoter region with APC methylation

Gene	Primer		Fragment size
APC	F	AGTGTGTGTAGAAGGATTTATTAATTGGG	380
	R	CAACCAACAACAATACCTAAAAACAACAT	

## Abbreviations

Abs: ameloblastoma
CIN: chromosome instability
ER2α: estrogen receptor
